# The CDC42 effector protein MRCKβ autophosphorylates on Threonine 1108

**DOI:** 10.1080/21541248.2018.1564472

**Published:** 2019-01-22

**Authors:** Mathieu Unbekandt, Sergio Lilla, Sara Zanivan, Michael F. Olson

**Affiliations:** a Cancer Research UK Beatson Institute, Glasgow, UK; b Institute of Cancer Sciences, University of Glasgow, Glasgow, UK; c Department of Chemistry and Biology, Ryerson University, Toronto, Canada

**Keywords:** kinase, CDC42, phosphorylation, MRCK

## Abstract

The CDC42 small GTPase is a major influence on actin-myosin cytoskeleton organization and dynamics, signalling via effector proteins including the *M*yotonic dystrophy *r*elated *C*DC42-binding protein *k*inases (MRCK) α and β. We previously identified Serine 1003 of MRCKα as a site of autophosphorylation, and showed that a phosphorylation-sensitive antibody raised against this site could be used as a surrogate indicator of kinase activity. In this study, a kinase-dead version of MRCKβ was established by mutation of the conserved Lysine 105 to Methionine (K105M), which was then used for mass spectrometry analysis to identify phosphorylation events that occurred in catalytically-competent MRCKβ but not in the kinase-dead form. A total of ten phosphorylations were identified on wild-type MRCKβ, of which the previously undescribed Threonine 1108 (Thr1108) was not found on kinase-dead MRCKβ K105M, consistent with this being due to autophosphorylation. Mutation of Thr1108 to non-phosphorylatable Alanine (T1108A) or phosphomimetic Glutamate (T1108E) did not affect the ability of MRCKβ to phosphorylate recombinant myosin light chain *in vitro*, or observably alter the subcellular localization of green fluorescent protein (GFP)-tagged MRCKβ expressed in MDA MB 231 human breast cancer cells. Although phosphorylation of Thr1108 did not appear to contribute to MRCKβ function or regulation, the identification of this phosphorylation does make it possible to characterize whether this site could be used as a surrogate biomarker of kinase activity and inhibitor efficacy as we previously demonstrated for Ser 1003 in MRCKα.

## Introduction

The actomyosin cytoskeleton is an interacting network of polymerized actin filaments (F-actin) and multi-molecular complexes of myosin heavy and light chains, which provide the structure and physical force that define cell shape and power morphological changes [1]. The forces generated by actomyosin contraction also contribute to dynamic processes including motility, division, and endocytosis at the cellular level, and muscle contraction at the tissue level. Aberrant regulation of actomyosin function may contribute to a range of human diseases or pathological conditions including cancer, hypertension and developmental disorders [2].

The small GTPase CDC42 plays essential roles in actin-myosin cytoskeleton regulation and cell motility [3,4] through effector proteins including *M*yotonic dystrophy *r*elated *C*DC42-binding protein *k*inases (MRCK) α and β [5,6]. MRCKα and MRCKβ are serine/threonine kinases, and are part of the larger AGC kinase family [7], including their next closest relatives ROCK1 and ROCK2 [8]. Several studies have reported that the MRCK and ROCK kinases act together to promote cancer cell motility and invasion, consistent with them having complementary roles in cytoskeleton regulation [9,10]. The MRCKα and MRCKβ kinase domains are approximately 85% identical and they share a common set of substrates, including phosphorylation of myosin II regulatory light chains (MLC) on Serine 18(S18), which leads to myosin activation and actin-myosin filament contraction. The discovery of potent MRCK inhibitors has enabled the characterization of the role of these kinases in cytoskeleton regulation, morphology and motility [11,12], as well as in skin cancer growth and glioma invasion [11,13].

Likely due to the close relatedness of the MRCK and ROCK kinase domains, no substrates specific to an individual kinase have been identified, with the exceptions of MRCKα autophosphoryation on Ser1003, ROCK1 on Ser1333 [14] and ROCK2 on Ser1366 [15]. Phospho-sensitive antibodies raised against these sites revealed that these autophosphorylation events were directly associated with kinase activity, indicating that the phosphorylation status of each residue can be used as a surrogate biomarker of the corresponding kinase’s activation state. Furthermore, immunohistochemical (IHC) analysis of breast cancer samples with the ROCK1 and ROCK2 autophosphorylation site antibodies revealed that nuclear ROCK2 activation was associated with increased metastasis and poor patient outcomes [16]. Similar IHC analysis of mouse models of skin cancer and human glioma specimens with an autophosphorylation-sensitive antibody indicated that increased MRCKα activity was associated with tumourigenesis [11,13].

In order to identify MRCKβ autophosphorylations, an approach similar to our previous study with MRCKα was employed [11], in which phosphorylation sites were identified by mass spectrometry (MS) that occurred on catalytically competent but not kinase-dead MRCKβ. In this study, we determined that active MRCKβ becomes phosphorylated on Thr1108, which does not occur on the kinase-dead protein. We investigated the function of this phosphorylation using *in vitro* kinase assays and analysis of protein subcellular by immunofluorescence microscopy.

## Results

### Expression of MRCKα and MRCKβ in human tissues

**Figure 1.**
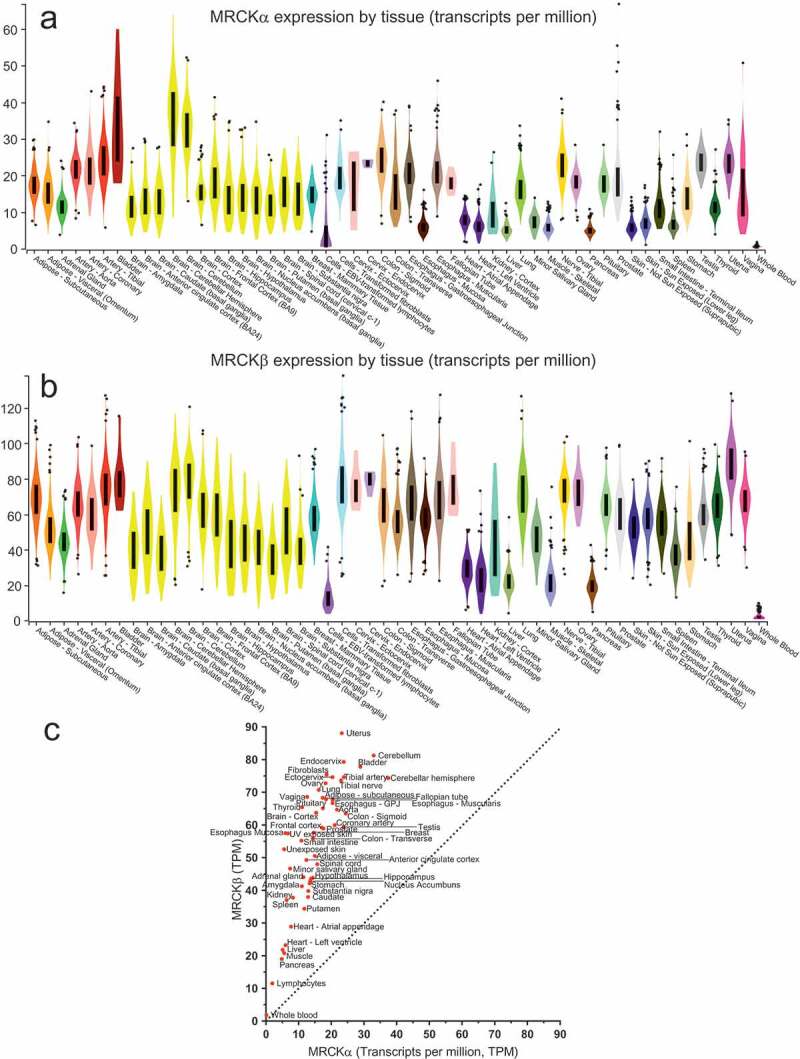
Gene expression of MRCKα and MRCKβ in human tissues. (a) The expression of MRCKα (Ensembl ENSG00000143776.14) and (b) MRCKβ (Ensembl ENSG00000198752.5) were deteremined by The Genotype-Tissue Expression (GTEx) project from 53 non-diseased tissue sites across 714 individuals (release v7). Expression values are shown in transcripts per million (TPM) in which possible isoforms were collapsed to a single gene. Box plots indicate upper and lower quartiles, outliers are ± 1.5 times the interquartile range. (c) Median gene expression values for MRCKα and MRCKβ were plotted to indicate their relative expression in each tissue. Dotted line has a slope = 1, points above it are expressed at relatively higher levels in MRCKβ relative to MRCKα.

Given the high degree of homology and overlap in biological functions between the two MRCK proteins, we wished to determine whether differences in the expression of the MRCKα (*CDC42BPA*, ENSG00000143776.14) and MRCKβ (*CDC42BPB*, ENSG00000198752.5) genes across human tissues might indicate that one or the other had a more prominent role in some contexts. Using the Broad Institute’s Genotype-Tissue Expression (GTEx) project portal (gtexportal.org/home/) examine tissue-specific gene expression from 53 non-diseased sites across 714 individuals, the patterns of MRCKα and MRCKβ expression were comparable across tissues (Figure 1(a,b)). However, the levels of MRCKβ expression, measured as specific transcripts per million (TPM) were higher than MRCKα in every tissue (Figure 1(c)). Deming regression analysis revealed that the slope of a fit line (2.98 ± 0.31) was significantly (p < 0.0001) different from 0, indicating that MRCKβ expression is approximately three-fold higher than MRCKα across tissues in humans. Assuming that the activity and function of the two MRCK proteins are equivalent, these data indicate that MRCKβ likely makes a relatively greater contribution than MRCKα in healthy and diseased tissues.

### Inactivation of MRCKβ by mutation of Lys105

**Figure 2.**
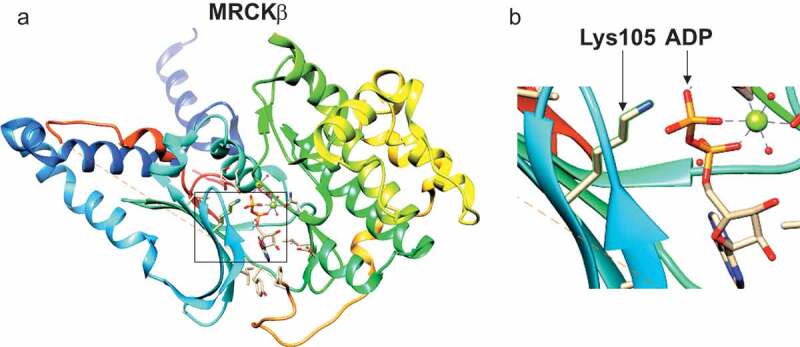
Position of MRCKβ Lys105. (a) The crystal structure of the kinase domain of MRCKβ in complex with ADP (PDB 4UAK) [10] was rendered with UCSF Chimera [28] to show the position of the Lys105 side chain relative to the ADP phosphates (boxed region). (b) The boxed region is shown at higher magnification to show the close proximity of the Lys105 side chain relative to the ADP phosphates.

**Figure 3.**
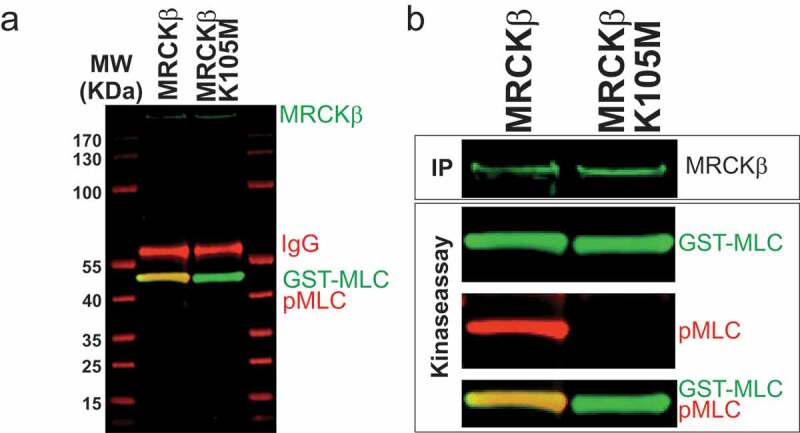
MRCKβ Lys105 is essential for kinase activity. (a) Western blot of immunoprecipitated myc-tagged MRCKβ and MRCKβ-K105M (green) that were placed in kinase buffer along recombinant GST-MLC (green). Probing with an antibody against the doubly phosphorylated Ser18/Thr19 MLC (pMLC, red) revealed that active MRCKβ phosphorylated MLC while no signal could be detected for full length MRCKβ K105M. The molecular weights (MW) of proteins in a MW ladder (red) are indicated in kDa. Immunoglobulin (IgG) from the immunoprecipitating antibody is revealed in red. (b) Individual panels of the immunoreactivity of immunoprecipitated (IP) myc-tagged MRCKβ proteins (green), and proteins in the *in vitro* kinase assay: GST-MLC (green), pMLC (red), MLC/pMLC overlay.

Members of the AGC kinase family have a conserved Lysine residue that is responsible for co-ordinating the α and β phosphates of ATP, which is essential for catalysis [17]. In MRCKα, Lys106 had previously been shown to be necessary for activity and substrate phosphorylation [18], which we also found to be necessary for autophosphorylation [11]. Our crystal structure of the MRCKβ kinase domain (PDB 4UAK) [10] (Figure 2(a)) placed the homologous Lys105 in close proximity to the ADP β phosphate (Figure 2(a) boxed region, Figure 2(b)). We mutated the MRCKβ Lys105 to Methionine (MRCKβ K105M) and investigated its ability to phosphorylate recombinant gluthathione-S-transferase (GST)-MLC. When myc-epitope tagged MRCKβ was immunoprecipitated (IP) from transfected HEK293 cells and assayed *in vitro* for the ability to phosphorylate GST-MLC on S18/T19 sites, the robust phosphorylation observed with MRCKβ was absent in the K105M mutant (Figure 3), indicating that Lys105 is essential for MRCKβ kinase activity.

### MRCKβ autophosphorylates on Thr1108

**Figure 4.**
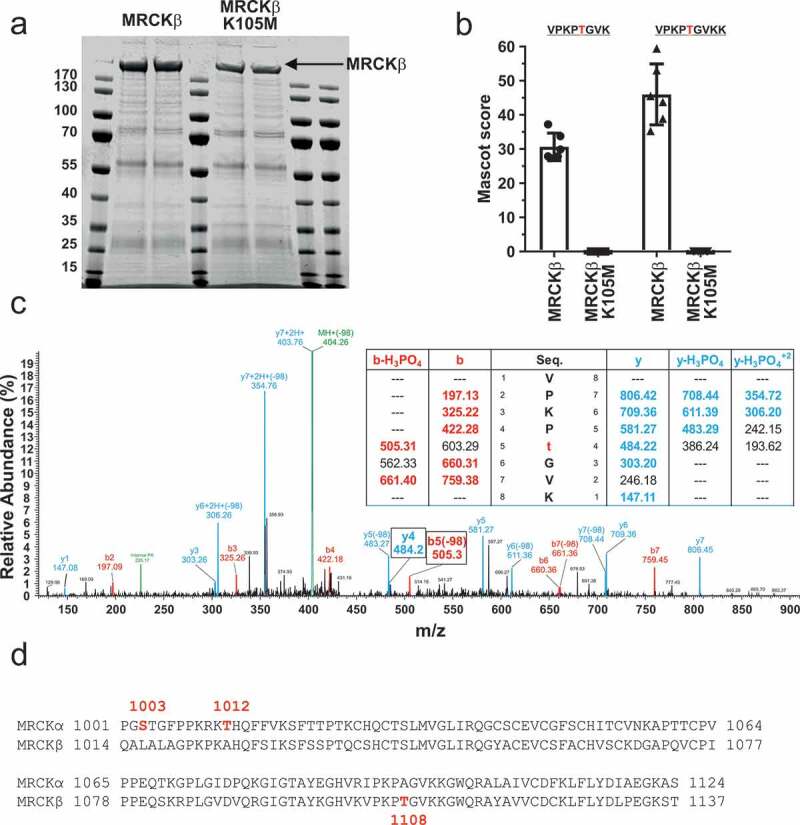
Identification of MRCKβ phosphorylations. (a) SDS-PAGE gels of immunoprecipitated MRCKβ and MRCKβ-K105M in duplicate were stained with Coomassie. Immunoprecipitations and subsequent mass spectrometry were performed in triplicate, giving a total of 6 separate replicates. (b) Mascot scores of the 6 replicates samples for the two overlapping peptides indicated that contain Thr1108 (red) from wild-type MRCKβ and kinase-dead MRCKβ K105M. (c) Positive ion MS/MS spectra and theoretical masses of b (red) and y (blue) fragmentation series of the MRCKβ peptide VPKPTGVK (1104–1111) containing phosphorylated Thr1108 (red), with the mass/charge peaks corresponding to Thr1108 indicated with boxes. (d) Alignment of the MRCKα and MRCKβ regions containing the autophosphorylation sites identified in each protein [11].

To identify MRCKβ autophosphorylation sites, mass spectrometry was used to identify amino acids modified by phosphorylation in full length active MRCKβ and in full length inactive MRCKβ-K105M. MRCKβ and MRCKβ-K105M were expressed in HEK293 cells, immunoprecipitated, and analyzed by mass spectrometry (Figure 4(a)). The experiment was repeated 3 times in duplicates resulting in 6 samples per condition. Two related tryptic fragments (amino acids 1104–1111 VPKP(pT)GVK and 1104–1112 VPKP(pT)GVKK) containing phosphorylated Thr1108 were observed in all 6 samples from wild-type MRCKβ but not from kinase-dead MRCKβ-K105M (Figure 4(b)). A total of 10 phosphorylation sites were identified (Table 1), of which 8 had been previously identified and are listed on the PhosphoSitePlus database (Thr423, Ser481, Thr676, Ser868, Ser1680, Ser1686, Ser1690 and Ser1693). Two sites were not previously identified (Thr1108 and Ser1683). An example MS/MS spectra of a human MRCKβ peptide (1104–1111 VPKPTGVK) containing phosphorylated Thr1108 is shown in Figure 4(c). The crystal structure of the MRCKβ kinase domain revealed that phosphorylation was not essential for an active conformation to be adopted, and consistent with this observation there were no phosphorylations detected in the amino-terminal kinase region. By inference, the previously unidentified Thr1108 phosphorylation is an autophosphorylation event since it was observed in active MRCKβ but not kinase-dead MRCKβ-K105M. Alignment of the regions adjacent to the previously identified MRCKα autophosphorylations (Ser1003 and Thr1012) [11] and the MRCKβ Thr1108 autophosphorylation revealed that none of these residues is conserved (Figure 4(d)).

**Table 1.** MRCKβ phosphorylation sites. MRCKβ phosphorylation sites identified by Mascot from Orbitrap data acquired in linear ion trap (CID in multistage activation) and/or higher energy collision dissociation (HCD). Peptide start-phosphorylation site-end positions are indicated for each peptide, with inferred autophosphorylation site in red. The reported Mascot ion score for an MS/MS match is −10Log(P) of the calculated probability P that a match between experimental data and database sequence is a random event. ND = not determined.

Peptide Sequence (Phosphorylation or Autophosphorylation Underlined)	Start-Phosphorylation-Stop Positions (Autophosphorylation)	MRCKβ Mascot Score (Mean, n = 6)	MRCKβ K105M Mascot Score (Mean, n = 6)
SIMQSN**t**LTKDEDVQR	417-T423-432	34.6	26.7
ALSN**s**NRDKEIK	477-S481-488	36.4	30.3
GAGA**t**LEHQQEISK	672-T676-685	68.9	68.3
**s**QKLDMSAR	868-S868-876	34.1	29.6
VPKP**t**GVK	1104-T1108-1111	30.6	ND
VPKP**t**GVKK	1104-T1108-1112	46.0	ND
HSTP**s**NSSNPSGPPSPNSPHR	1676-S1680-1696	ND	35.9
HSTPSNS**s**NPSGPPSPNSPHR	1676-S1683-1696	ND	41.5
HSTPSNSSNP**s**GPPSPNSPHR	1676-S1686-1696	44.7	42.7
HSTPSNSSNPSGPP**s**PNSPHR	1676-S1690-1696	113.2	112.9
HSTPSNSSNPSGPPSPN**s**PHR	1676-S1693-1696	90.0	88.3
HSTPSNSSNP**s**GPP**s**PNSPHR	1676-S1686-S1690-1696	61.1	54.4
HSTPSNSSNPSGPP**s**PN**s**PHR	1676-S1690-S1693-1696	61.4	41.1

### Thr1108 phosphorylation does not regulate MRCKβ activity

**Figure 5.**
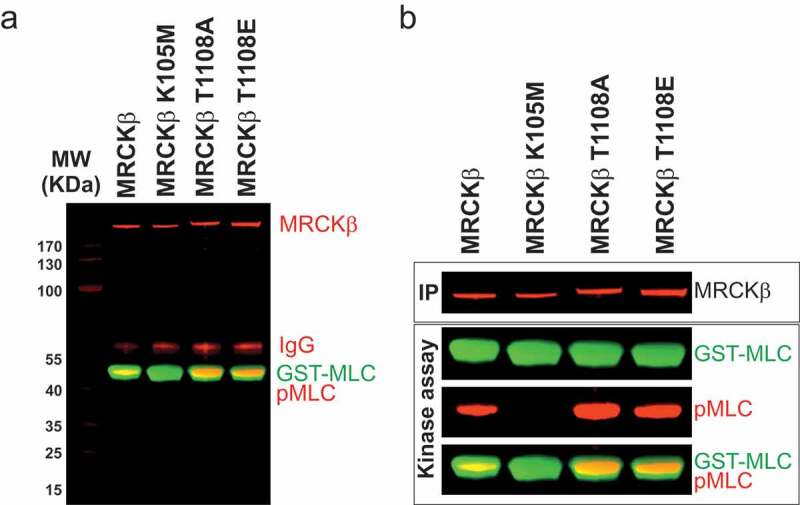
Thr1108 phosphorylation is not required for substrate phosphorylation. (a) Western blot of immunoprecipitated myc-tagged MRCKβ, MRCKβ-K105M, MRCKβ-T1108A and MRCKβ-T1108E (red) that were placed in kinase buffer along recombinant GST-MLC (green). Probing with pMLC antibody (red) revealed that active MRCKβ, MRCKβ-T1108A and MRCKβ-T1108E all comparably phosphorylated MLC while no signal could be detected for kinase-dead MRCKβ K105M. MW ladder wieghts (red) indicated in kDa. IgG from the immunoprecipitating antibody in red. (b) Individual panels of the immunoreactivity of immunoprecipitated (IP) myc-tagged MRCKβ proteins (red), and proteins in the *in vitro* kinase assay: GST-MLC (green), pMLC (red), MLC/pMLC overlay.

Previously published analysis of MRCKα regulation suggested that an internal region containing two coiled-coil domains (amino acids 658 to 930) negatively regulated kinase activity [18], raising the possibility that similarly positioned phosphorylations might regulate activity. Having identified Thr1108 as a putative MRCKβ autophosphorylation, we investigated the role of this phosphorylation on kinase activity towards a substrate protein by changing Thr1108 to a non-phosphorylatable Alanine (T1108A) or to phosphomimetic Glutamic acid (T1108E). As previously observed, wild-type MRCKβ robustly phosphorylated recombinant GST-MLC and MRCKβ K105M did not, while both MRCKβ T1108A and MRCKβ T1108E phosphorylated substrate to a comparable extent (Figure 5(a,b)). These observations indicate that phosphorylation of Thr1108 is not required for activity, nor does it negatively affect substrate phosphorylation.

### Thr1108 phosphorylation does not regulate subcellular localization

**Figure 6.**
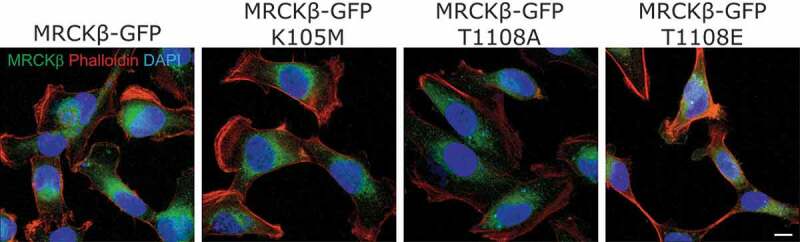
Subcellular localization of GFP-tagged MRCKβ is not affected by mutation of Thr1108. Confocal microscope images of MDA-MB-231 D3H2LN cells expressing MRCKβ-GFP, MRCKβ-GFP K105M, MRCKβ-GFP T1108A or MRCKβ-GFP T1108E (green). Samples were stained with Texas-Red conjugated phalloidin (red) to visualize filamentous actin and DAPI (blue) to localize nuclei. Scale bar corresponds to 10 µm.

MRCKα and MRCKβ have been reported to be typically cytoplasmic, with a proportion being translocated to, or concentrated at, the plasma membrane upon activation [19,20]. To investigate the potential role of Thr1108 phosphorylation on MRCKβ cellular localization, we used fluorescence microscopy to examine the distribution of wild-type MRCKβ with a carboxy-terminal green fluorescent protein tag (MRCKβ-GFP), and of kinase-dead MRCKβ-GFP K105M, non-phosphorylatable MRCKβ-GFP T1008A and phosphomimetic MRCKβ-GFP T1008E in transfected MDA-MB-231 D3H2LN human breast cancer cells [21]. No obvious differences in MRCKβ distribution were observed when the Thr1108 phosphorylation site was mutated to Alanine or to Glutamic acid, indicating that this site does not have a major role in determining MRCKβ subcellular localization (Figure 6).

## Discussion

In this report, we determined that the MRCKβ Lys105 in the ATP binding pocket (Figure 2) is essential for kinase activity, mutation of Lysine to Methionine resulted in the protein being catalytically inactive (Figure 3), similar to previous observations on the essential role of Lys106 in MRCKα [11] of the protein and that mutation of this amino acid renders the kinase dead.

By comparing the patterns of phosphorylation between wild-type and kinase-dead MRCKβ, we identified 9 common phosphorylations and 1 that uniquely occurred in wild-type but not kinase-dead MRCKβ (Figure 4), consistent with this being an autophosphorylation event. By mutating Thr1108 to a non-phosphorylatable Alanine (T1108A) or phosphomimetic Glutamic Acid (T1108E), we determined that phosphorylation of this site is not required for kinase activity (Figure 5), nor does it contribute substantially to determining MRCKβ subcellular localization (Figure 6). This phosphorylation has not been previously identified in studies curated in the PhosphoSitePlus database.

We previously showed that the autophosphorylation of MRCKα on Ser1003 could be used as a surrogate indicator of kinase activity. An antibody against the phosphorylated MRCKα Ser1003 revealed elevated levels of active kinase in genetic and chemically-induced mouse skin tumours, and also provided evidence that topical application of a selective MRCK small molecule inhibitor successfully blocked kinase activity [11]. We attempted to raise a similar rabbit polyclonal phospho-specific antibody against the MRCKβ Thr1108 site using the peptide VPKP(pT)GVKKGWQRAC. The resulting antibody showed phospho-specificity when assayed by ELISA using immobilized phosphorylated and unphosphorylated peptides (data not shown), but the antibody did not perform adequately well for Western blotting of cell lysates or for immunohistochemistry (data not shown). By reporting the putative MRCKβ Thr1008 autophosphorylation, we hope this will encourage others to attempt to develop such an antibody, which would be useful to investigate MRCKβ biology and development of MRCK inhibitors. Given that MRCKβ expression is higher than MRCKα in every tissue examined (Figure 1), it could be argued that the means to determine the level of MRCKβ activity in diseases conditions or following inhibitor treatment would be more informative than the S1003 phosphorylation-sensitive antibody that we were previously able to successfully generate and validate [11].

## Materials and methods

### Plasmids

The original source for the MRCKβ plasmids was the pEGFP-N1-Cdc42BPB plasmid deposited by Naoki Mochizuki in Addgene (#50759) [22]. pEGFP-N1- MRCKβ-K105M was generated by site directed mutagenesis of wild-type pEGFP-N1-MRCKβ using the Quickchange II XL kit (Agilent Technologies) according to manufacturer’s recommendations using the following primers:

K105M forward: 5ʹ-atactgaacgaatttatgcaatgatgatcctcaacaagtgggagatgc-3ʹ

K105M reverse: 5ʹ-gcatctcccacttgttgaggatcatcattgcataaattcgttcagtat-3ʹ

The pcDNA-Myc-MRCKβ and pcDNA-Myc-MRCKβ-K105M plasmids were generated by inserting MRCKβ sequences from pEGFP-N1-MRCKβ and pEGFP-N1-MRCKβ-K105M into a pcDNA-myc construct using restriction enzymes Kpn1 and BamH1.

The pEGFP-N1-MRCKβ-T1108A, pEGFP-N1-MRCKβ-T1108E, pcDNA-Myc-MRCKβ-T1108A and pcDNA-Myc-MRCKβ-T1108E plasmids were generated using the Q5 sited directed mutagenesis kit (New England Biolabs) from pcDNA-Myc-MRCKβ using the following primers:

T1108A forward: 5ʹ-CCCAAAGCCCGCGGGGGTGAAGA-3ʹ

T1108A reverse: 5ʹ-ACCTTGACATGGCCTTTGTAGGCTG-3ʹ

T1108E forward: 5ʹ-CCCAAAGCCCGAGGGGGTGAAGA-3ʹ

T1108E reverse: 5ʹ-ACCTTGACATGGCCTTTG-3ʹ

### MLC protein production

*E. coli* BL21 bacteria were transformed with pGEX2T-MLC and grown on ampicillin plates (50 µg/mL). A single colony was picked and cultured overnight at 37°C in 100 mL of LB broth supplemented with ampicillin (100 µg/mL). The culture was diluted 1 in 10 (30 mL into 300 mL of LB broth + ampicillin) and cultured for 1 hour at 37°C. IPTG was applied at 50 µM for 3 hours, then cultures were centrifuged at 4000 rpm for 15 minutes at 4°C, and the pellet was lysed in TBS +5 mM MgCl_2_ + 1 mM DTT +1 mM PMSF. The lysates were sonicated 3 times for 1 minute on ice and then centrifuged at 10,000 rpm for 10 minutes at 4°C. The supernatant was collected and used for *in vitro* MLC phosphorylation assays.

### HEK293 cell culture, transfection and immunoprecipitation

HEK293 cells were routinely cultured in DMEM+ 10% FCS + L-glutamine at 37°C. 1 × 10^6^ HEK293 cells were plated per 10 cm culture plate, then the following day medium was replaced with OPTIMEM. After 1 hour, cells were transfected in OPTIMEM with 10 μg of pcDNA-Myc-MRCKβ, pcDNA-Myc-MRCKβ-K105M, pcDNA-Myc-MRCKβ-T1108A or pcDNA-Myc-MRCKβ-T1108E as indicated using Fugene HD (Promega). After 6 hours, medium was replaced with DMEM 10%FCS + L-glutamine. Two days after transfection, cells were placed on ice, washed in ice cold PBS and lysed in 1 mL of ice cold lysis buffer (TBS + 1 mM EDTA + 1% Triton-X100 + 1 mM PMSF + 1X cOmplete Protease inhibitor (Roche) + 20 mM NaF + 20 mM β-glycophosphate + 0.2μM Na_3_VO_4_ + 20 μg/mL Aprotinin). Lysates were incubated on a rotating wheel for 30 minutes at 4°C before centrifugation at 13,200 rpm for 10 minutes at 4°C, then supernatants were collected. Lysates were incubated with anti-Myc agarose beads (Sigma, A7470) on a rotating wheel for 2 hours at 4°C. The beads were then washed 3 times in lysis buffer by successive centrifugations at 3000 rpm for 1 minute at 4°C. For mass spectrometry and western blots, beads were boiled at 95°C in 1% SDS for 5 minutes, centrifuged at 3000 rpm for 2 minutes and supernatants collected.

### Western blots

Cell lysates and immunoblot analysis were performed as described in [12]. The following antibodies were used: rabbit anti-pMLC2 Thr18/Ser19 (Cell Signaling Technology, 3674), MRCL3/MRLC2/MYL9 (Santa Cruz Biotechnology, sc-28329), anti-DMPK (MANDMN1) [23,24], and mouse anti-Myc tag (Cell Signaling Technology, #2276).

### MLC assays

For *in vitro* MLC phosphorylation assays, MRCKβ protein bound to anti-Myc beads after immunoprecipitation were re-suspended in 95 μL of kinase buffer (20 mM Tris HCl pH 7.4, 0.5 mM MgCl_2_, 0.01% Tween 20 and 1 mM DTT) with 2 μL of 5 mM ATP and 3 μL of recombinant GST-MLC and incubated with constant agitation at 30°C for 1 hour. To stop the reaction, 100 μL of boiling 2% SDS was added to the samples and the reactions were incubated for 5 minutes at 95°C. The samples were centrifuged at 3000 rpm for 2 minutes and supernatants were collected for western blots.

### Mass spectrometry

Full length MRCKβ and full length MRCKβ-K105M were expressed in HEK293 cells and immunoprecipitated as described above. Samples were run on SDS-PAGE, and stained using InstantBlue Coomassie protein stain (Expedeon). The bands containing MRCKβ were excised and digested with trypsin according to a previously described procedure [25]. The tryptic digests obtained were separated by nanoscale C18 reverse-phase liquid chromatography using an EASY-nLC II (Thermo Fisher Scientific) coupled to a Linear Trap Quadrupole (LTQ) Orbitrap Velos mass spectrometer (Thermo Fisher Scientific). The eluted peptides were injected into the mass spectrometer via a nanoelectrospray ion source (Sonation). The mass spectrometer was operated in positive ion mode and used in data-dependent acquisition. Fragmentation was performed on the top ten most intense ions using both available fragmentation modes: collision energy dissociation (CID, using a multistage activation option) and higher energy collision dissociation (HCD) in two separated acquisitions.

Raw data obtained were processed with MaxQuant version 1.5.5.1 [26]. Andromeda peak list files (.apl) generated were converted to Mascot generic files (.mgf) using APL to MGF Converter (wehi.edu.au/people/andrew-webb/1298/apl-mgf-converter). MGF files were searched using Mascot (Matrix Science, version 2.4.1), querying the UniProt [27] *Homo sapiens* database (09/07/2016; 92,939 entries), plus an in-house database containing common proteomic contaminants and the sequence of kinase-dead MRCKβ. Mascot was searched assuming the digestion enzyme trypsin allowing for two miscleavages with a fragment ion mass tolerance of 0.1 Da and a parent ion mass tolerance of 15 ppm. The iodoacetamide derivative of cysteine was specified in Mascot as a fixed modification. Oxidation of methionine and phosphorylation of serine, threonine and tyrosine were specified in Mascot as variable modifications. The MS/MS data of phosphopeptides were manually curated with Xcalibur Qual Browser version 2.2 (Thermo Scientific), and the MS-Product utility of Protein Prospector v5.12.4 (prospector.ucsf.edu/) was used to generate theoretical product ions fragmentation series.

Raw data, msms.txt files from MaxQuant and Mascot DAT files were imported into Skyline to build a library of MRCKβ peptides. Extracted ions chromatograms (XICs) of the 3 main isotopic peaks (30 K resolution at 400 m/z) of precursor ions from unmodified and phosphorylated peptides of MRCKβ that carried 2+ and 3+ charges were used for quantification of autophosphorylation sites.

### MDA-MB-231 D3H2LN cell culture, transfection and immunofluorescence

MDA-MB-231 D3H2LN cells were cultured in DMEM + 10% FCS + L-glutamine. Cells were plated at 10^5^ cells per well of a 6 well plate, then the following day medium was replaced with OPTIMEM. After 1 hour, cells were transfected in OPTIMEM with 3.3 μg of pEGFP-N1-MRCKβ, pEGFP-N1-MRCKβ-T1108A or pEGFP-N1-MRCKβ-T1108E as indicated using Fugene HD (Promega). After 6 hours, medium was replaced with DMEM + 10% FCS + L-glutamine. Two days after transfection, cells were washed in PBS and fixed in 4% paraformaldehyde for 15 minutes. Cells were washed twice in PBS, permeabilized for 15 minutes in 0.5% Triton X-100 in PBS, washed twice in PBS and incubated for 1 hour in 1% BSA in PBS. Cells were incubated for 1 hour at room temperature with Texas-red conjugated phalloidin (Molecular Probes, Invitrogen). Cells were washed and coverslips were mounted using Vectashield mounting medium containing DAPI.
